# Physical activity when riding an electric-assisted bicycle with and without cargo

**DOI:** 10.3389/fspor.2023.1179043

**Published:** 2023-06-30

**Authors:** Jørgen Jerstad Martnes, Elling Bere

**Affiliations:** ^1^ Department of Sports Science and Physical Education, University of Agder, Kristiansand, Norway; ^2^ Department of Health and Inequalities & Centre for Evaluation of Public Health Measures, Norwegian Institute of Public Health, Oslo, Norway

**Keywords:** active transport, electric-assisted bicycle, cargo bike, exercise intensity, physical activity

## Abstract

**Background:**

Regular physical activity provides several health benefits, and active transport is a convenient way to implement physical activity in everyday life. However, bikes’ lack of possibilities to carry cargo is a limitation. E-cargo bikes can help overcome barriers to cycling and increase levels of active transport while still providing the option to carry cargo such as groceries and children. As such, E-cargo bikes have a greater potential for being a substitute for cars, but relevance is not known as no study has assessed the energy expenditure and time used using E-cargo bikes with considerable cargo.

**Objectives:**

The aim of this study is to compare time spent riding and exercise intensity when (1) riding an electric-assisted bicycle with cargo (30 kg) and without cargo and (2) driving a car.

**Method:**

This study has a randomised crossover design. Eleven participants (six women) were recruited through convenience sampling. The participants traversed through a 4.5 km route with three different forms of transportation: an electric-assisted bicycle (E-bike) with 30 kg cargo, an E-bike without cargo, and a car. Oxygen uptake was measured with a portable oxygen analyser (Metamax 3B), and time spent cycling was measured on site by the test leader using a stopwatch.

**Results:**

Riding an E-bike with cargo was slightly slower than riding an E-bike without cargo (11.8 vs. 11.1 min, *p* = 0.017) and driving a car (8.8 min, *p* = 0.002). There was no significant difference in exercise intensity between E-bikes with and without cargo but riding an E-bike with cargo entailed significantly higher exercise intensity compared to driving a car [4.9 metabolic equivalents of task (METs) vs. 1.4 METs, *p *≤ 0.001].

**Conclusions:**

E-biking with cargo was rather similar in time spent and exercise intensity to E-biking without cargo, and not much slower than driving a car. Using E-cargo bikes, therefore, appears a good alternative to driving a car when in need of carrying things such as grocery bags and children, resulting in increasing physical activity and, at the same time, decreasing greenhouse gas emissions.

## 1. Background

Human beings have always carried things, be it food, water, babies, or belongings (1). Carrying has thus been an important source of daily physical activity (PA) throughout history. Today, we walk less and carry less of our things with us as we lean on motorised vehicles, primarily cars, for transport.

While we let technology do physical everyday tasks for us, we become increasingly inactive. Inactivity is a major public health issue, both globally (2) and in Norway (3). Modern society and infrastructure is built around motorised vehicles and roads, which makes it practically challenging to travel and carry the things we need without a car (4). In addition to the negative impact on public health, increased use of motorised vehicles leads to more greenhouse gas emissions (5).

In recent years, electric-assisted bicycles (E-bikes) have emerged as an environmental-friendly mode of transportation (6, 7) that can get more people cycling (8, 9). Previous research shows that using an E-bike entails at least moderate-intensity physical activity (10–14) while simultaneously contributing to overcoming common barriers to cycling (9, 15).

Transporting goods decreases the chance of commuting by bicycle on a given day (16). For example, transporting children makes women less likely to choose the bicycle as a transportation mode (17) and going to other places, such as the store or kindergarten, before or after work is a negative predictor for cycling (18). E-cargo bikes can potentially solve challenges related to transporting goods and children and get more people to choose active transportation (AT) rather than driving a car (19). Cargo bikes can provide benefits to the transport of children and gear and are shown to have the potential to further increase active transportation and decrease car travel compared to regular bicycles (20). Cargo bikes come in various shapes and sizes, now usually with electric assist capacity. Research indicates that longtail E-bikes may be more feasible for transporting goods and children as compared to conventional bikes with trailers and human-powered longtails for untrained individuals (21).

Previous studies (10, 13, 14) have found E-bikes to be faster and have lower exercise intensity compared to conventional bicycles. The literature on physical activity when using E-bikes with cargo, however, is scarce. The existing literature on E-cargo bikes concentrates more on commercial transport, but the greatest potential for a shift from cars to bicycles when transporting goods lies in private use due to the short distance trips and light cargo (22). To investigate whether E-cargo bikes are a feasible transportation option to increase PA, the present study will compare time spent riding and exercise intensity when (1) riding 13 an electric-assisted bicycle with cargo (30 kg) and without cargo and (2) compared to driving a car.

## 2. Methods

### 2.1. Research design

The present study is an experimental study with a randomised crossover design, where data were collected during an intervention in Kristiansand, Norway. The trial consisted of a route mimicking a trip from work or school to either kindergarten or a grocery store. The participants traversed through the route with three different forms of transportation; an E-bike with cargo, an E-bike without cargo, and a car. All of the participants used the E-bike with and without cargo, and then drove a car depending on whether or not they had a driving license and a car available. Data collection took place during the fall of 2021, from September 11 to September 25.

Participants first underwent a baseline screening consisting of anthropometric measurements, answering a questionnaire assessing background information and travel habits, and, finally, calculating their resting metabolic rate (RMR). Subsequently, they traversed through the route on an E-bike with cargo, an E-bike without cargo, and potentially a car, in randomised succession. While cycling/driving through the route, participants had their oxygen uptake (VO_2_) measured with a portable oxygen analyser, and time spent was measured on site by the test leader. In total, baseline measurements and field tests took between 1.5 and 2 h for each participant.

**Figure 1:**
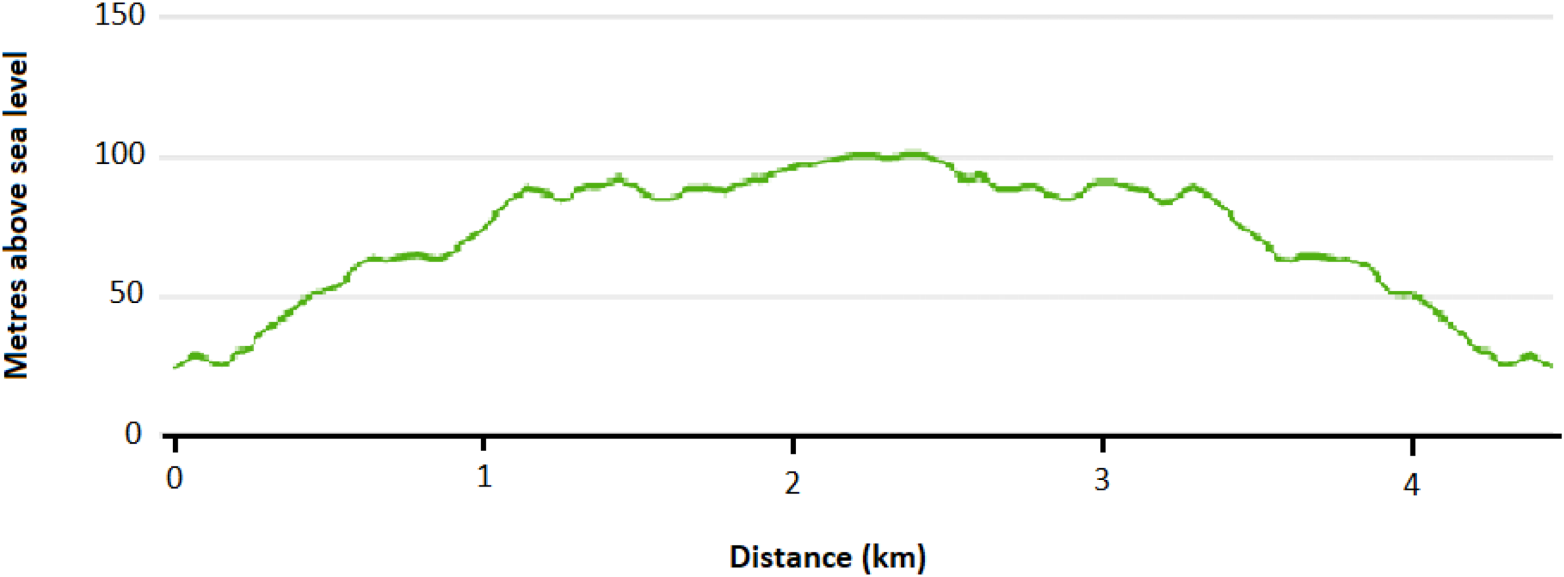
Altitude profile of the field-trial route.

The E-bike, a Riese & Muller Multicharger Mixte GT light longtail bike with a wheel size of 26" and bike weight of 24.9 kg, was used on maximal electrical power. It has a Performance Line CX (Gen3) motor with a maximal speed of 25 km/h with an active engine, and a Bosch PowerTube 500 Vertical, 36 V, 13.4 Ah/500Wh* battery. The cargo weight was 30 kg in total, mimicking potentially either four shopping bags weighing 7.5 kg each or a child weighing 30 kg. The route started and ended at the same spot and was approximately 4.5 km, which coincides well with the average distance of travels done by bicycle in Norway in 2020 (Opinion AS, 2021) and previous studies on E-bikes and exercise intensity (10, 11, 13). The altitude of the point of departure and destination was 24 m above sea level, and the highest altitude of the route was 100 m above sea level (see Figure 1). The first half of the route had several steep hills, while the second half (back the same way) was primarily downhill.

### 2.2. Study sample

The study population were adults in Norway. A convenience sample was picked from students and employees at the University of Agder. In total, 12 people were selected to participate in the study and 11 (5 men and 6 women) were included in the final analysis. Data from one participant were excluded. This was due to the data showing unrealistically low values for VO_2_ and was therefore considered not valid. All 11 participants used the E-bike with cargo and without cargo, and 6 participants also drove a car. All the participants were non-smokers.

Participants had to be able to read and understand Norwegian, as well as be able to ride a bike to participate in the study. Reported serious injury or illness was the only criteria for exclusion. Informed consent was obtained from all the participants.

Research clearance was obtained from the Faculty Ethical Committee at the Faculty of Health and Sport Sciences of the University of Agder and from the Norwegian Social Science Data Services (NSD).

### 2.3. Measurements

#### 2.3.1. Field tests

A portable oxygen analyser (MetaMax 3B-R2, CORTEX Biophysik GmbH, Leipzig, Germany) was used to measure the participants’ oxygen uptake. While cycling or driving, the participants wore the MetaMax in a vest and a breathing mask covering their mouth and nose. Prior to use, the system was turned on for at least 30 min and then calibrated before every test. First, barometric pressure was measured and the MetaMax was calibrated. Second, the gas analyser was calibrated by using a reference gas (15% O_2_, 6% CO_2_) and then the calibration was verified against ambient air. Finally, a volume calibration was performed according to the manufacturer's recommendations. When initiating a new test, the MetaMax adjusted the sensors by calibrating against ambient air for a second time before the measurement could start. Time spent cycling by each participant was measured on site by the test leader using a stopwatch.

#### 2.3.2. Baseline measurements

Participants answered a questionnaire for baseline characteristics and to provide consent to participation in the study. The questionnaire assessed travel and activity habits and relevant background information such as sex, date of birth, and country of birth, together with information determining eligibility for inclusion. Height and weight (in light clothing without shoes) were measured, and RMR was calculated. RMR was obtained by indirect calorimetry with an oxygen analyser (MetaMax) according to best practice methods (23), using a standardised protocol. Participants lay supine on a massage table while wearing the MetaMax and a breathing mask. After calibration, measurement was started, and a timer was set for 15 min. Anything between 7 and 20 min was sufficient for attaining data to calculate RMR, provided the first 5 min are deleted (23). Therefore, only the last 10 min of the measurement were included in the calculation.

Exercise intensity was measured as absolute values, using metabolic equivalents of task (METs). MET is a physiological measure expressing the intensity of physical activities and is defined as the ratio of the working metabolic rate to a person's resting metabolic rate (24). One MET represents the resting metabolic rate and is the energy equivalent expended by an individual while seated at rest, usually expressed as ml O_2_/kg/min (25). While traversing through the routes, oxygen uptake was measured with a portable oxygen analyser (MetaMax) and a breathing mask. METs were calculated by dividing each participant's average oxygen uptake when traversing through the given route, by their RMR.

### 2.4. Statistical analysis

IBM SPSS statistics (version 25) was used to conduct all statistical analyses. Descriptive statistics is presented as mean and SD. Paired samples *t*-test was used to test differences in time spent cycling, VO_2_, exercise intensity (METs), MET-minutes, and time spent in moderate to vigorous physical activity (MVPA) and vigorous physical activity (VPA) between E-biking with and without cargo and E-biking with cargo and driving a car. METs, MET-minutes, and time spent in MVPA and VPA were calculated both from measured RMR and estimated RMR (1 MET = 3.5 ml/min/kg).

## 3. Results

Baseline characteristics for the participants are presented in Table 1. The participants had a mean age of 32 years, a mean height of 175 cm, and a mean weight of 74 kg. The mean BMI was 24, and the mean RMR was 3.9 ml O_2_/min/kg.

**Table 1:** Participant characteristics.

	Mean	SD	Min–max
Age (years)	32	9	24–47
Height (cm)	175	10	164–199
Weight (kg)	74	11	60.0–87.5
BMI	24.0	2.4	20.8–29.2
RMR (O_2_ ml/min/kg)	3.9	0.5	3.4–4.7

The mean time spent was 11.1 min when riding an E-bike without cargo, 11.8 min for an E-bike with cargo, and 8.8 min for a car. Riding the E-bike with cargo took significantly more time than both E-bike without cargo (*p* = 0.017) and car (*p* = 0.002). Mean oxygen uptake was 18.6, 19.1, and 5.5 ml O_2_/min/kg when using the E-bike without cargo, E-bike with cargo, and car, respectively. Riding an E-bike with cargo resulted in a significantly higher VO_2_ than driving a car (*p* = 0.001). When using the measured RMR, the mean exercise intensity was 4.7 METs when riding the E-bike without cargo, 4.9 METs when riding the E-bike with cargo, and 1.4 METs when driving the car. Riding the E-bike with cargo resulted in a significantly higher exercise intensity than driving the car (*p* = 0.028), but there was no significant difference between E-bike with and without cargo (*p* = 0.294). Using the measured RMR, the mean MET-minutes for an E-bike without cargo was 52.0, for E-bike with cargo it was 57.5, and for a car it was 12.0. E-bike with cargo had significantly higher MET-minutes than E-bike without cargo (*p* = 0.041) and car (*p* = 0.028) (see Table 2).

**Table 2:** Mean (SD) and mean difference (95% CI) in time spent cycling and VO_2_, and MET, MET-minutes, time spent cycling and time spent in MVPA and VPA based on 1 MET—3.5 ml O_2_/min/kg and measured RMR, between riding an e-bike with and without cargo, and riding an e-bike with cargo and driving a car.

	E-bike without cargo	E-bike with cargo	Car	Mean difference E-bike without cargo − E-bike with cargo	*p*-value	Mean difference E-bike with cargo − car	*p*-value
Time (min)	11.1 (1.4)	11.8 (1.3)	8.8 (0.8)	0.7 (0.2 to 1.2)	0.017*	2.4 (1.4–3.4)	0.002*
VO_2_	18.6 (3.6)	19.1 (3.6)	5.5 (0.6)	0.6 (−0.6 to 1.7)	0.282	14.0 (9.2–18.9)	0.001*
METs	4.7 (0.8)	4.9 (0.8)	1.4 (0.1)	0.1 (−0.1 to 0.4)	0.294	3.4 (2.5–4.4)	<0.001*
MET-minutes	52.0 (7.9)	57.5 (11.8)	12.0 (1.4)	5.6 (1.3 to 9.9)	0.016*	41.2 (32.7–49.6)	<0.001*
MVPA (min)	9.5 (1.1)	9.9 (1.7)	0 (0)	0.5 (−0.2 to 1.1)	0.747	9.3 (8.1–10.6)	<0.001*
VPA (min)	2.9 (2.5)	3.0 (2.4)	0 (0)	0.1 (−1.9 to 2.1)	0.921	2.2 (0.02–4.3)	0.048*
Estimated METs	5.3 (1.0)	5.5 (1.0)	1.6 (0.2)	0.2 (−0.2 to 0.5)	0.282	4.0 (2.6–5.4)	0.001*
Estimated MET-minutes	58.4 (9.2)	64.4 (12.3)	13.9 (1.8)	6.0 (1.3 to 10.7)	0.018*	48.3 (34.6–62.0)	<0.001*
Estimated MVPA (min)	10 (1.1)	10.5 (1.2)	0 (0)	0.5 (0.1 to 1.0)	0.025*	10.2 (9.4–11.0)	<0.001*
Estimated VPA (min)	3.6 (2.3)	4.5 (2.5)	0 (0)	0.8 (−0.1 to 1.8)	0.082	4.2 (1.2–7.1)	0.015*

CI, confidence interval

* *p* ≤ 0.05.

When using an estimated RMR of 3.5 ml O_2_/min/kg, the mean exercise intensity was 5.3 METs when riding an E-bike without cargo, 5.5 METs when riding an E-bike with cargo, and 1.6 METs when driving a car. The estimated mean MET-minutes were 58.4, 64.4, and 13.9 for E-bike without cargo, E-bike with cargo, and car, respectively.

Car driving registered 0 min in MVPA. E-biking without cargo had a mean of 9.5 min spent in MVPA and 2.9 in VPA, while E-biking with cargo had a mean of 9.9 min in MVPA and 3 in VPA. MVPA-minutes accounted for 86% and 84% of time spent cycling for E-bike without cargo and E-bike with cargo, respectively.

**Figure 2:**
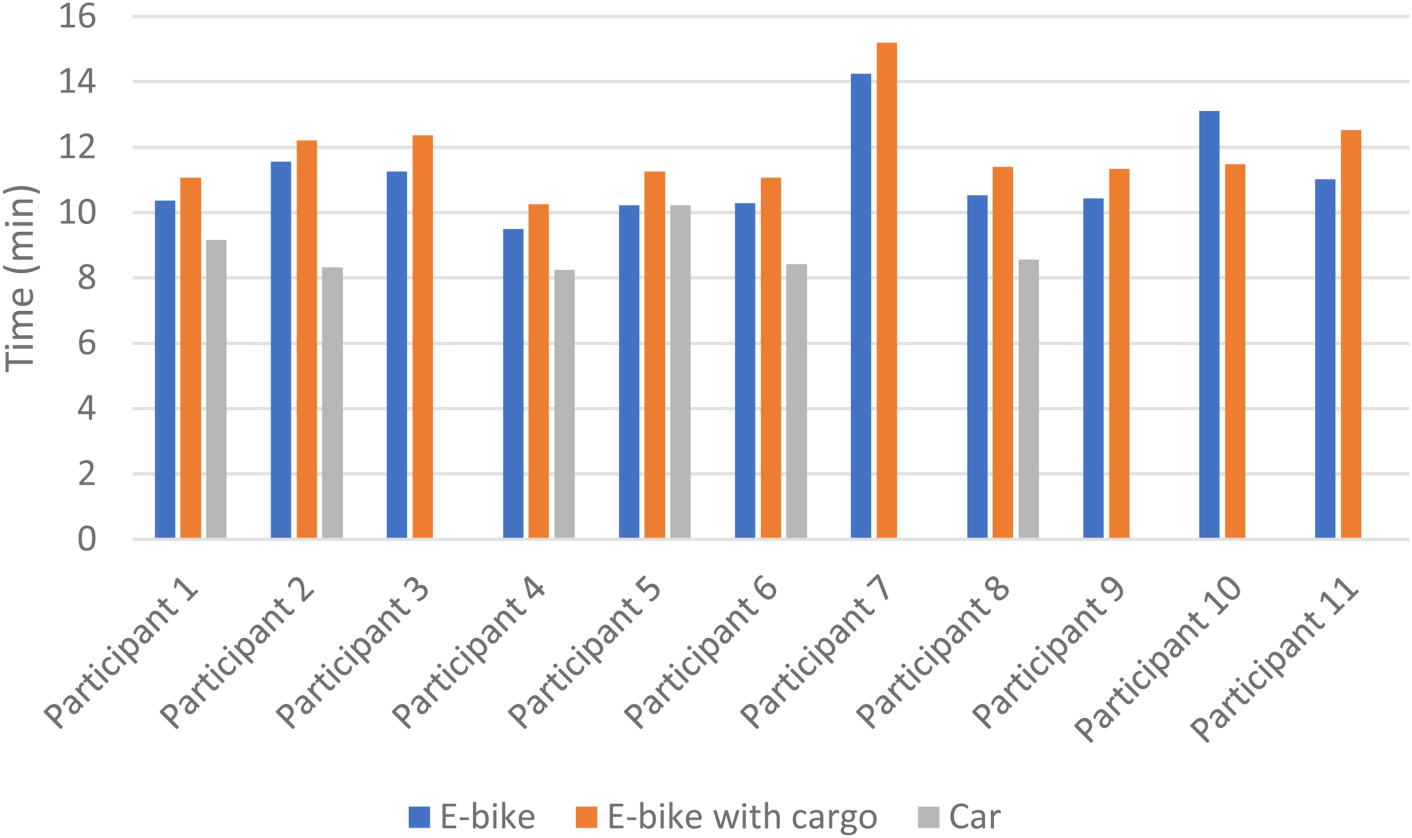
Individual differences in time (min) spent riding E-bike with and without cargo and driving a car.

**Figure 3:**
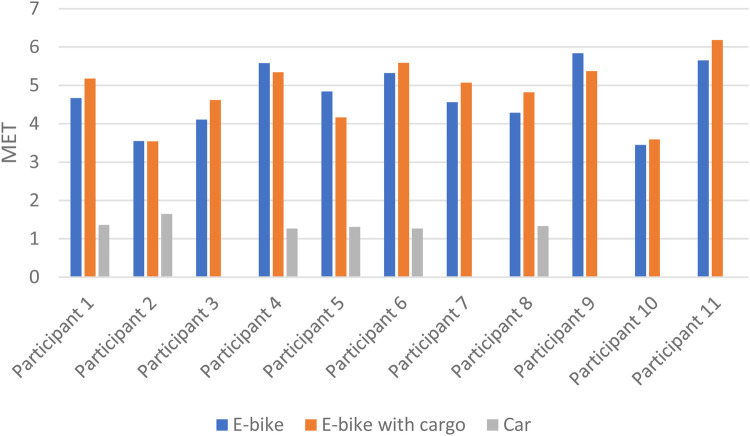
Individual differences in METs for E-bike with and without cargo and car.

**Figure 4:**
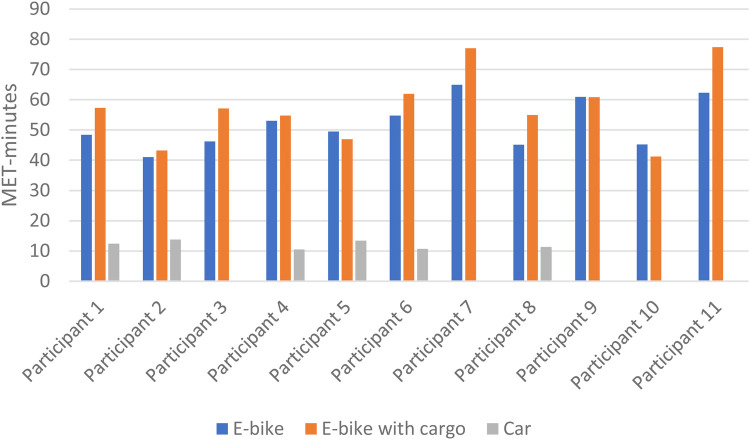
Individual differences in MET-minutes for E-bike with and without cargo and car.

Some variation between the participants was found in time spent cycling/driving, but all except one (participant 10) had the same ranking for the three different transportation modes, with the car as the fastest and the E-bike with cargo as the slowest (Figure 2). Figures 3, 4 show considerable individual variations in METs and MET-minutes for E-bike with and without cargo. The range from lowest to highest MET was 3.5–5.8 for E-bike without cargo and 3.5–6.2 for E-bike with cargo. The range from lowest to highest MET-minutes was 41.0–65.0 for E-bike without cargo and 41.2–77.4 for E-bike with cargo. Regarding METs, the ranking of E-bike without cargo, E-bike with cargo, and car was similar for most participants, but three of them (participants 4, 5, and 9) were separated from the rest, having higher METs when using the E-bike without cargo than the E-bike with cargo. Similarly, three participants (5, 9, and 10) had higher MET-minutes when using the E-bike without cargo than the E-bike with cargo.

## 4. Discussion

In this study, time spent cycling/driving and exercise intensity were compared between E-bikes with and without cargo and E-bike with cargo and car; the aim being to examine the physical activity as well as the feasibility of E-biking with cargo. E-bike without cargo was found to be somewhat faster than the E-bike with cargo (0.7 min, 6%), but there was no significant difference in exercise intensity between them (4.7 vs. 4.9 METs). The car was faster than the E-bike with cargo (3 min, 25%) and had a significantly lower exercise intensity (3.8 METs, 71%). Furthermore, riding the E-bike with cargo entailed significantly more MET-minutes than both the E-bike without cargo (5.5 MET-minutes, 11%) and the car (45.5 MET-minutes, 379%).

E-biking has previously been found to be faster (10, 13) and less physically strenuous (26) than conventional cycling, and the present study shows that adding considerable cargo (30 kg) does little to time spent cycling and exercise intensity. Changing transportation mode from car to E-cargo bike can, however, increase exercise intensity to nearly four times as high (1.4 vs. 4.9 METs) while being only somewhat slower and maintaining the option to transport goods or children.

In the present study, most of the time spent cycling with the E-bike, both with and without cargo, was MVPA, supporting and building upon the findings of previous studies (10, 13, 14, 27). The amount of total cycling time spent in MVPA with E-bike with cargo was slightly lower than without cargo (86% vs. 84%). The reason for this might be that participants spent more time cycling downhill with the E-bike with cargo because they felt more unstable due to the extra weight, thereby spending more time in the part of the route demanding less intensity. Cycling on the E-bike with cargo registered a mean of 2.9 min in VPA, while E-bike without cargo registered a mean of 3 min in VPA (25% vs. 26%). The route consisted of primarily upward hills towards a turning point and downward hills back to the start/finishing point, so it would be logical to assume that the VPA minutes happened during the first half of the route. In a different setting with fewer downward hills, the number of VPA minutes would likely increase to similar numbers as in the study by Gojanovic et al. (10), who found 47.1% of the time spent cycling to be in VPA on a route with less downhill.

In Norway, adults are recommended to reach at least 150 min of MVPA or 75 min of VPA per week (28), which accounts for 450 MET-minutes. Mean MET-minutes was 52 for E-bike without cargo and 57.5 for E-bike with cargo in the present study. Thereby, to reach the PA recommendations, one would need nine and eight trips with E-bike without cargo and E-bike with cargo, respectively. Considering that the amount of cycling increases for people using E-bikes (8, 9) and cargo bikes (20), E-cargo bikes may be a good option to maintain a PA level consistent with the national recommendations.

The results from this study imply that an E-cargo bike can be an effective tool to make more people switch the transportation mode from car to bicycle, also when there is a need to carry cargo. At the same time, the results show that E-cargo bikes entail PA of at least moderate intensity, which provides several health benefits (29–31). E-cargo bikes may thus be a good option to improve public health in a society where the majority of people use cars as a transportation mode, also for shorter distances (32).

To the author's knowledge, only one previous study has measured physical activity when cycling on an E-bike with considerable cargo (33) found that energy expenditure increased by less than 5% on E-cargo bikes when adding 16 and 32 kg cargo, compared to three times as much for a conventional cargo bike. This coincides well with the results from the present study, where no difference was found in exercise intensity between E-bikes with and without cargo and demonstrates how much more physically demanding conventional cargo bikes can be compared to E-cargo bikes.

The strengths of this study include the use of a real-life setting and direct measurement of oxygen uptake. The route was chosen to simulate travel from work or school to either kindergarten or the store, and the altitude profile was meant to be representative of the hilly terrain of Norway. The MetaMax 3B is found to be reliable and stable in field studies but overestimates VO_2_, VCO_2_ (carbon dioxide production), and V_E_ (expired ventilation) in moderate and vigorous PA when compared to the Douglas bag method (gold standard) and a second validated criterion (Oxycon Pro) (34).

Another strength of the present study is the use of measured RMR, which is considered more precise than using an estimated RMR. The estimated RMR of 3.5 ml O_2_/kg/min does not take individual factors such as weight status, fitness, sex, and age into consideration and has been shown to overestimate peoples resting metabolic rate (35). The present study, however, found a mean RMR of 3.9 ml O_2_/kg/min. Several factors may have caused the higher mean RMR of the present study. First of all, the small sample size may have affected the measurements. However, in their study in 2017, Berntsen et al. (14) found a lower median RMR in their participants (3.0 ml O_2_/kg/min), having a similarly small sample size. Best practice methods for the measurement of RMR (23) were followed in the present study, but behaviour such as physical activity and caffeine-intake among the participants prior to participating may also have affected their RMR. The use of different gas analysers may also be the cause of the difference in RMR in this study compared to Berntsen et al. (14). In their study, Berntsen et al. used a stationary gas analyser (Oxycon Pro), while the MetaMax 3B was used in the present study. Macfarlane and Wong (33) found that the VO_2_ values measured by MetaMax 3B were significantly higher compared to the measurement of Oxycon Pro during rest. Although measured RMR is considered more precise, the conclusion on the research question of the present study would have been the same with estimated RMR.

There are several limitations to the present study. Firstly, the small sample size and convenience sample may have reduced the generalizability of the results. Since the study involved physical activity, it might have primarily attracted physically fit individuals to participate. Another limitation is that GPS was not used during the trial. Using a GPS when traversing through the route could have illustrated the differences in intensity in the different segments of the route more precisely. External factors such as weather conditions and traffic could not be controlled for; however, no trials were done when it was raining, following the manufacturers’ guidelines for the protection of the MetaMax 3B gas analyser. Participants performed all three field tests on the same day and had only a small break between trials, but the order of transportation mode used was randomised, limiting the effect potential fatigue from the first round may have had on the results.

## 5. Conclusion

In this experiment, over a distance of 4.5 km, cycling on an E-bike with considerable cargo (30 kg) was almost as fast as cycling on an E-bike with no cargo, and driving a car was only 25% faster. The intensity of cycling with cargo was only somewhat higher than without cargo, but about four times as high as driving a car. E-biking with cargo might, therefore, reintroduce the option of attaining physical activity through carrying things in modern life and thus contribute to reaching national recommendations for PA.

## Data Availability

The raw data supporting the conclusions of this article will be made available by the authors, without undue reservation.
